# Remote ischemic preconditioning attenuates cardiopulmonary bypass-induced lung injury

**DOI:** 10.1371/journal.pone.0189501

**Published:** 2017-12-12

**Authors:** Xiaona Zhou, Runzhu Jiang, Yucai Dong, Lifeng Wang

**Affiliations:** 1 Department of Anesthesiology, Guizhou Province’s People Hospital. Guiyang, Guizhou, China; 2 Department of Ansthesiology, Women & Children’s Health Care Hospital of Linyi, Linyi, Shandong, China; 3 Department of Rehabilitation, Linyi People’s Hospital, Linyi, Shandong, China; 4 Department of Anesthesiology, Linyi People’s Hospital, Linyi, Shandong, China; University of PECS Medical School, HUNGARY

## Abstract

The use of cardiopulmonary bypass (CPB) in cardiac surgeries is known to induce pathological changes in vital organs such as lungs. Remote ischemic preconditioning (RIPC) is a protective strategy that has shown to be able to reduce tissue damage related to ischemia-reperfusion injury (IRI). The current study seeks to evaluate the beneficial effects of limb RIPC on lung tissues and function in a rat CPB model. RIPC, which consisted of three cycles of 5-min ischemia and subsequently 5-min reperfusion, was induced in the hind limbs of the animals via a tourniquet. Bronchoalveolar lavage (BAL) fluid analysis and hematoxylin and eosin staining revealed that limb RIPC could significantly attenuate CPB-induced pulmonary injury, as evidenced by a combination of lower total BAL protein content, less severe alveolar wall thickening and reduced intra-alveolar neutrophil infiltration. Consistently, RIPC was also found to improve the proliferation capacity of the bronchioalveolar stem cells isolated from the lung tissues in rats subjected to surgical procedure with CPB. These beneficial effects translated into significantly improved lung function. Further investigation suggested that RIPC could up-regulate the serum levels of several anti-inflammatory cytokines such as interleukin (IL)-4 and 10, which might play a role in its pulmonoprotective effects. Taken together, the current study provided convincing evidence that limb RIPC could be a useful strategy for minimizing CPB-induced organ injuries in patients undergoing CPB surgery.

## Introduction

It is widely known that the use of cardiopulmonary bypass (CPB) in cardiac surgeries can lead to a series of pathological changes with varying severities, including ischemia-reperfusion injury (IRI) and systemic inflammatory response syndrome, in vital organs such as heart and lungs [1]. Combined, these symptoms contribute to a significant part of post-operative complications and mortality for common cardiopulmonary diseases that require surgical intervention, such as many types of congenital cardiac defects and valvular heart diseases. The mechanism for the development of CPB-triggered heart and lung injuries is complex and multifactorial. There have been studies suggesting that the exposure of blood to artificial surfaces of CPB components could activate neutrophils and monocytes through multiple signal cascades, leading to widespread inflammatory response throughout the circulatory system [2]. It is worth noting that these processes are also facilitated by various proinflammatory cytokines [3]. The activated neutrophils then migrate to and are eventually sequestered in the lung, where they can inflict damage to local tissues by secreting various proteases [4]. In addition, there is also evidence that ischemia in the lung could also be a contributing factor to CPB-induced pulmonary injury [3].

Limb remote ischemic preconditioning (RIPC) is a clinical strategy to protect tissues from IRI [5]. The general idea is that the controlled induction of nonlethal and transient ischemia in one organ or tissue, such as skeletal muscles, can confer ischemic resistance in a distant vital organ or tissue. Although the molecular mechanism underlying the protective effects of limb RIPC remains elusive, results obtained from earlier studies have suggested that such intervention could lead to the generation of inflammatory cytokines and other small regulatory factors that can spread via the circulatory system to the target area where they would mitigate IRI-induced injury [6]. The clinical effects of limb RIPC have been evaluated in animal models and humans on a wide range of pathologies. Cheung et al. have reported that non-invasive limb RIPC could induce protective effects on myocardial tissues in children undergoing repair of congenital heart defects [7]. Olguner et al. demonstrated that limb RIPC could be used to mitigate pulmonary injury in a murine model of unilateral lower limb ischemia reperfusion [8]. Li and colleagues studied and experimentally confirmed the pulmonoprotective benefits of limb RIPC in a randomized control trial of 216 patients that received lung resection under anesthesia induced by Propofol-remifentanil [9]. In comparison, there have been very few studies on whether RIPC could also protect lung tissues from CPB-induced injury.

In the current study, we aim to investigate whether RIPC could confer pulmonary protection in rats that undergo CPB surgery. We also seek to examine the mechanism that underlies such protective effects. We found significant alleviation of pulmonary injury and improvement of lung function in rats subjected to CPB and limb RIPC treatment, compared to those that underwent CPB but not RIPC. Furthermore, RIPC was shown to result in increased expression of interleukin (IL)-4 and IL-10 in the serum samples collected from the subjects, suggesting that the enhancement of anti-inflammatory pathways might play a key mechanistic role.

## Materials and methods

### Rat CPB model

To construct the rat CPB model, 45 adult male Sprague-Dawley rats weighing 350–450 g and aged 6–8 weeks were obtained from the Animal Center of Shandong University, China. All subsequent animal experiments were in compliance with the National Institute of Health guidelines and approved by the Animal Care and Use Committee of Linyi Hospital, China. The rats were housed in an air-conditioned room and fed a standard diet. Prior to the surgical procedures, the rats were randomly divided into three equal groups (15 in each group), including a sham (control) group, a CPB group and an RIPC treatment group.

Both the CPB group and the treatment group were individually annulated and underwent CPB surgery. Briefly, the rats were intraperitoneally injected with 2% butaylone at a dose of 50 mg/kg body weight and maintained under anesthesia with additional butaylone. A 24-gauge inflow catheter and a 22-gauge outflow catheter were inserted into the right carotid artery and right femoral vein, respectively. Additionally, a 22-gauge catheter was inserted into the right femoral artery for blood monitoring and sampling. The CPB circuit consisted of a 20-mL sterile venous reservoir, a roller pump and a membrane oxygenator with around 4-mL pre-charge and a gas exchange area of 0.05 m^2^ (Xijing Medical Co. Ltd, Xi’an, Shaanxi, China), all of which were connected to each other through sterile silicone tubes. The circuit was primed with a 10-mL mixed solution comprising 1 mL of 250 IU/kg heparin, 0.5 mL of 5% sodium bicarbonate, 3.5 mL of Lactated Ringer’s Solution and 5 mL of 6% hydroxyethyl starch in 0.9% sodium chloride. The flow rate was then gradually increased to 100 mL/kg body weight/min and maintained at this level for 60 min, where the mean arterial pressure stayed relatively stable in the range of 60 to 80 mmHg. The body (rectal) temperature of each rat was monitored and maintained between 36.5–38.5°C by placing a warm blanket on its back and a heat lamp nearby. After the CBP procedure was completed, the outflow cannula was removed and the right femoral vein was ligatured. The remaining priming solution was slowly infused into the carotid artery until stable circulation was achieved. The inflow catheter was subsequently removed and the carotid artery was ligatured, followed by intramuscular administration of 2000 U/kg body weight of penicillin.

The anesthetized rats in the treatment group then were subjected to three cycles of 5-min ischemia followed by 5-min reperfusion in their right hind limbs via a tourniquet. The animals in the sham group were annulated but did not receive surgery or treatment. As a result, the sham group served as a negative control that should represent normal baseline pulmonary function, whereas the CPB group was a positive control indicative of unmitigated bypass-induced lung injury.

### Measurement of pulmonary function

Evaluation of pulmonary function in rats was performed 24 h after the CPB using an invasive pulmonary function device (Buxco Research Systems, Wilmington, NC, USA) based on a previously described procedure with minor modifications [10]. Briefly, the rats were anesthetized, subjected to tracheal intubation and then placed on the device. An average respiration rate of 50 breaths/min was applied. The following parameters were recorded by the built-in software, including the total lung capacity (TLC), airway resistance (R_aw_) and dynamic lung compliance (C_dyn_).

### Post-mortem analysis

At the end of pulmonary function tests, rats were sacrificed by exsanguination under anesthesia. The lung was dissected from the thorax and bronchoalveolar lavage (BAL) was performed. Differential leucocyte counts were performed on the obtained BAL fluid samples.

The fluid samples were centrifuged and total protein was determined by using the BCA Protein Assay Kit (Beyotime, Jiangsu, China). The levels of granulocyte-macrophage colony stimulating factor (GM-CSF), IL-1α, IL-1β, IL-2, IL-4, IL-10, IL-12, tumor necrosis factor-α (TNF-α) and interferon-γ (IFN-γ) in BAL fluid were quantified using Cytokine Rat 10-Plex Panel for the Luminex platform (Thermo Fisher Scientific, Waltham, MA, USA).

### Lung wet-to-dry weight ratio

The right lung of each rat was removed following the euthanasia. After the removal of the trachea and esophagus by blunt dissection, the wet weight of the lung was determined. Subsequently, the lung was incubated at 60°C for 3 to 4 days to remove all moisture, after which its dry weight was measured and the wet-to-dry weight ratio was calculated.

### Pressure-volume (P-V) curve

P-V curves were obtained based on a previously reported protocol [11]. In brief, 20 mL normal saline containing 5 mM EDTA was injected into the vena cava to flush the lung vasculature. A 16-gauge angiocatheter (BD, Franklin Lakes, NJ, USA) was inserted into the trachea through the neck incision, immobilized with a suture, and then connected to a syringe pump (World Precision Instruments, Sarasota, FL, USA) with an in-line pressure sensor (Flowplus, Intertronics, Oxfordshire, England). The lung P-V curves were monitored and recorded on a ViscoTec Flowscreen Evaluation Unit (Intertronics, Kidlington, Oxfordshire, UK). The flow rate of the injected air was around 25 mL/min and the direction of the pump was reversed when the pressure exceeded 40 cm H_2_O or started to increase dramatically [12].

### Histological assessment

The left lung was dissected and fixed. Lung damage was assessed by quantitative stereological analysis. In brief, pulmonary circulation was rinsed with phosphate buffer and perfused with 4% paraformaldehyde. Then, ligation was performed between the pulmonary artery and trachea, followed by the inflation of the lung with 4% paraformaldehyde. After the fixation, the lung was embedded in paraffin and cut into 7-μm-thick slices, which were then mounted on slides and stained with hematoxylin and eosin (H&E). Images were acquired randomly at 20× and 40× magnification. Six high-power fields were randomly selected in each photograph and lung injury scores were calculated based on the algorithm proposed by Faller et al [13].

### Isolation and culture of brochioalveolar stem cells (BASC)

BASCs were isolated from the lungs following a previously described protocol [14]. Dulbecco’s Modified Eagle Medium: Nutrient Mixture F12 (DMEM-F12), 10% FCS-Joklik’s MEM, PE-conjugated anti-Sca-1, FITC-conjugated anti-CD34, PE-CY5-conjugated anti-CD45 and PE-CY7-conjugated anti-CD31 were obtained from Thermo Fisher Scientific (Waltham, MA, USA). Dispace was purchased from Roche (Basel, Switzerland) and 0.1% protease type-XIV was procured from Sigma-Aldrich (St. Louis, MO, USA). Cell sorting was performed on a FACSAria I flow cytometer (BD, Franklin Lakes, NJ, USA).

The BASCs isolated from different animal groups were monitored by the xCELLigence RTCA DP station (ACEA Biosciences, San Diego, CA, USA). Roughly 5*10^3^ cells were added to each well of a 96-well sample plate containing 100 μL MEM with 10% fetal bovine serum. The presence of the cells could modify the local ionic environment around the electrodes located at the bottom of the well, causing the electrode impedance to increase. The amount of increase was correlated to the total number of cells attached to the electrodes and the total overlapping areas between the cells and the electrodes.

### Statistical analysis

Continuous variables are expressed as mean ± SD. Statistical analysis was performed with the aid of SPSS (version 20.0; SPSS Inc, Chicago, IL, USA) using either ANOVA or Student-Newmann-Keuls multiple comparison test. P<0.05 was considered statistically significant.

## Results

### Limb RIPC significantly attenuates lung injury induced by surgical procedure with CPB

**Fig 1:**
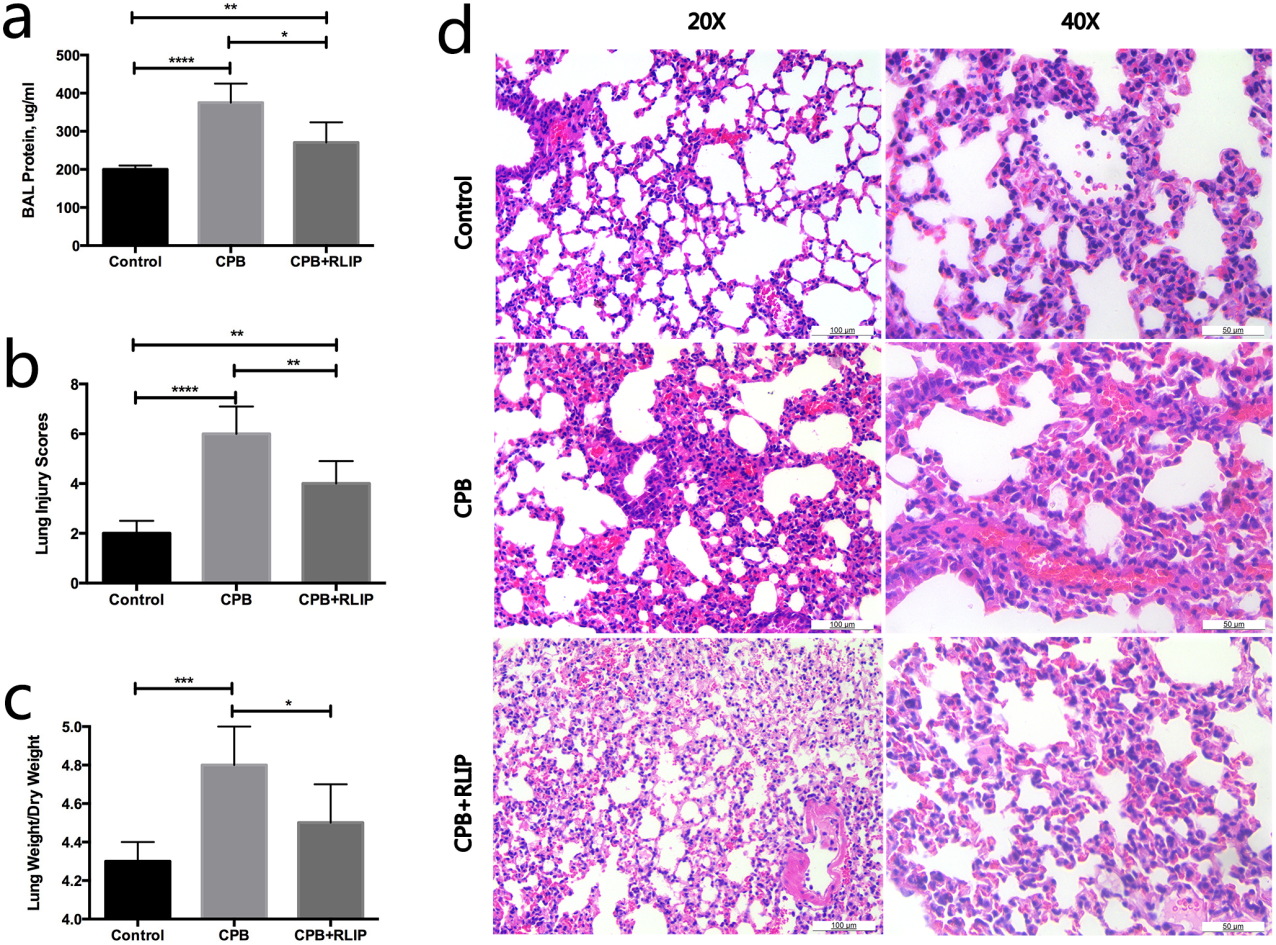
Assessment of lung injury in different animal groups. Comparison of the total BAL protein levels (a), lung injury scores (b) and lung weight-to-dry weight values (c) among the three groups. (d) H&E staining of lung tissues obtained from different groups at 20× and 40× magnification. *, P ≤ 0.05; **, P ≤ 0.01; ***, P ≤ 0.001; ****, P ≤ 0.0001.

We began our study by comparing the extents of lung injury in the three experiment groups. As expected, the total BAL protein concentration in rats increased significantly as a result of CPB surgery (Fig 1a), which indicated increased permeability of the alveolar-capillary membrane, and concomitantly, pulmonary injury. This was further confirmed by the H&E staining results that showed intra-alveolar infiltration of neutrophils and thickening of the alveolar walls in animals of the CPB group compared to the control subjects (Fig 1b and 1d). However, it was also apparent that the treatment with limb RIPC could significantly mitigate the CPB-induced lung injuries as reflected by the reduction in the total BAL protein level and the alleviation of the abovementioned histological abnormalities (Fig 1a, 1b and 1d). Similarly, we measured the lung wet/dry weight ratios in the three groups as an indicator of pulmonary edema. As illustrated in Fig 1c, rats in the CPB group exhibited a significant elevation in the lung wet-to-dry weight ratio compared to the control, suggesting an abnormal accumulation of fluids in their lungs. In comparison, the same ratio in the treatment group showed a significant decline in comparison to that of the CPB group (Fig 1c). These data combined demonstrated that limb RIPC could attenuate CPB-induced lung injury.

### Limb RIPC significantly mitigates loss of lung function caused by CPB

**Fig 2:**
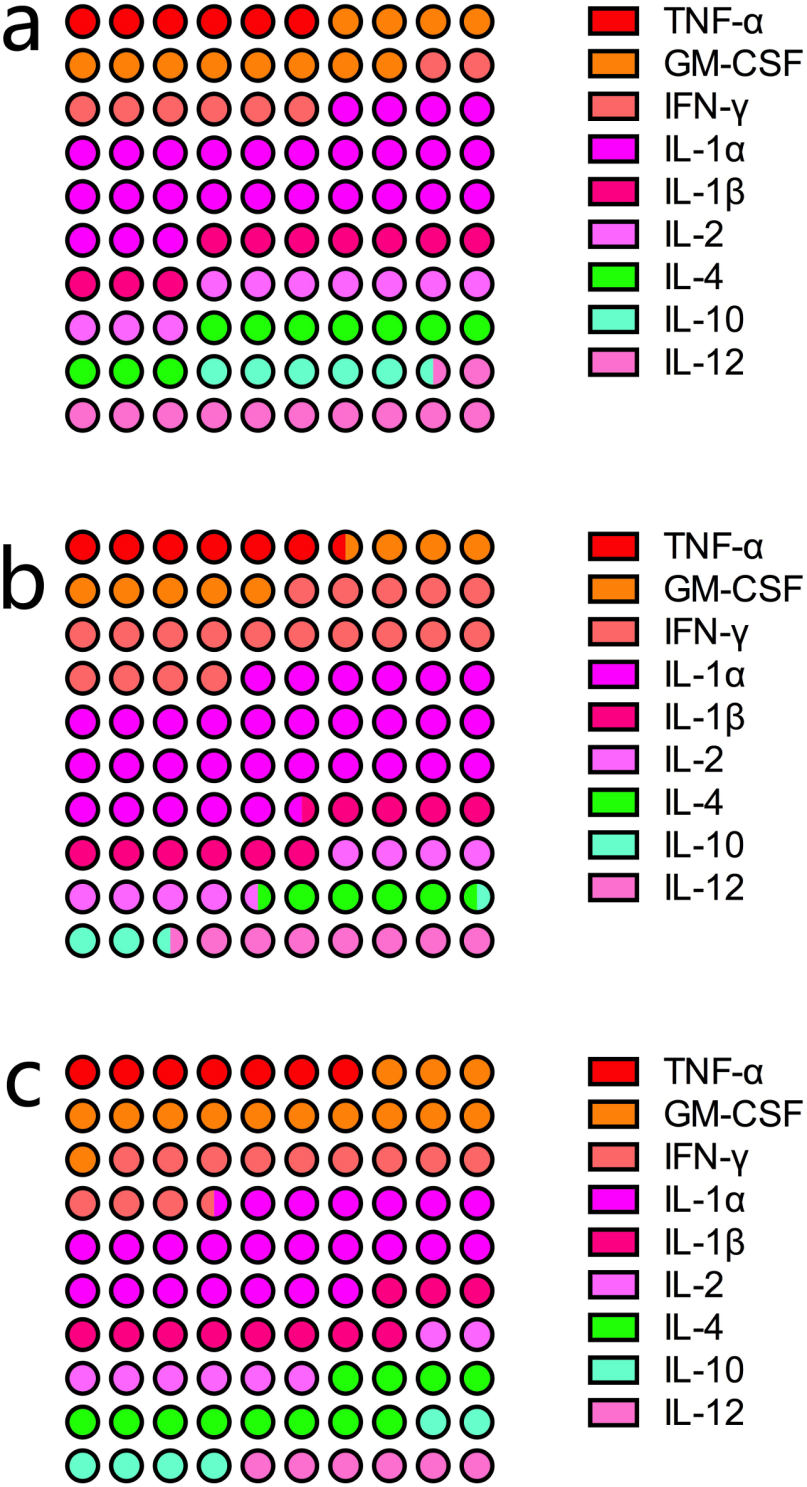
Assessment of lung function in different animal groups. Comparison of TLV (a), R_aw_ (b) and C_dyn_ (c) among the three groups. (d) Comparison of the P-V curves of the three groups. *, P ≤ 0.05; **, P ≤ 0.01.

We then investigated whether the RIPC-treated rats would also show improvement in their lung function. Indeed, the average TLC dropped significantly from 1.3 mL in the control group to 1.0 mL in the CPB group (Fig 2a). Meanwhile, the rats subjected to limb RIPC showed a partial restoration of TLC to 1.2 mL (Fig 2a). On the other hand, CPB was also found to significantly increase R_aw_ and lower C_dyn_ of the animal subjects, both of which served as further evidence of impaired lung function (Fig 2b and 2c). In these cases, the average R_aw_ and C_dyn_ of the treatment group displayed intermediate values between those of the control and of the CPB group (Fig 2b and 2c). Measurement and comparison of the lung P-V curves also corroborated the above findings as a downward shift was observed for the CPB group in comparison to the control, the trend of which was partially reversed in the treatment group (Fig 2d). Taken together, we concluded that the mitigation of pulmonary injury by limb RIPC in rats that underwent CPB surgery could also significantly ameliorate their lung function.

### Limb RIPC improves the viability and proliferation of BASCs

**Fig 3:**
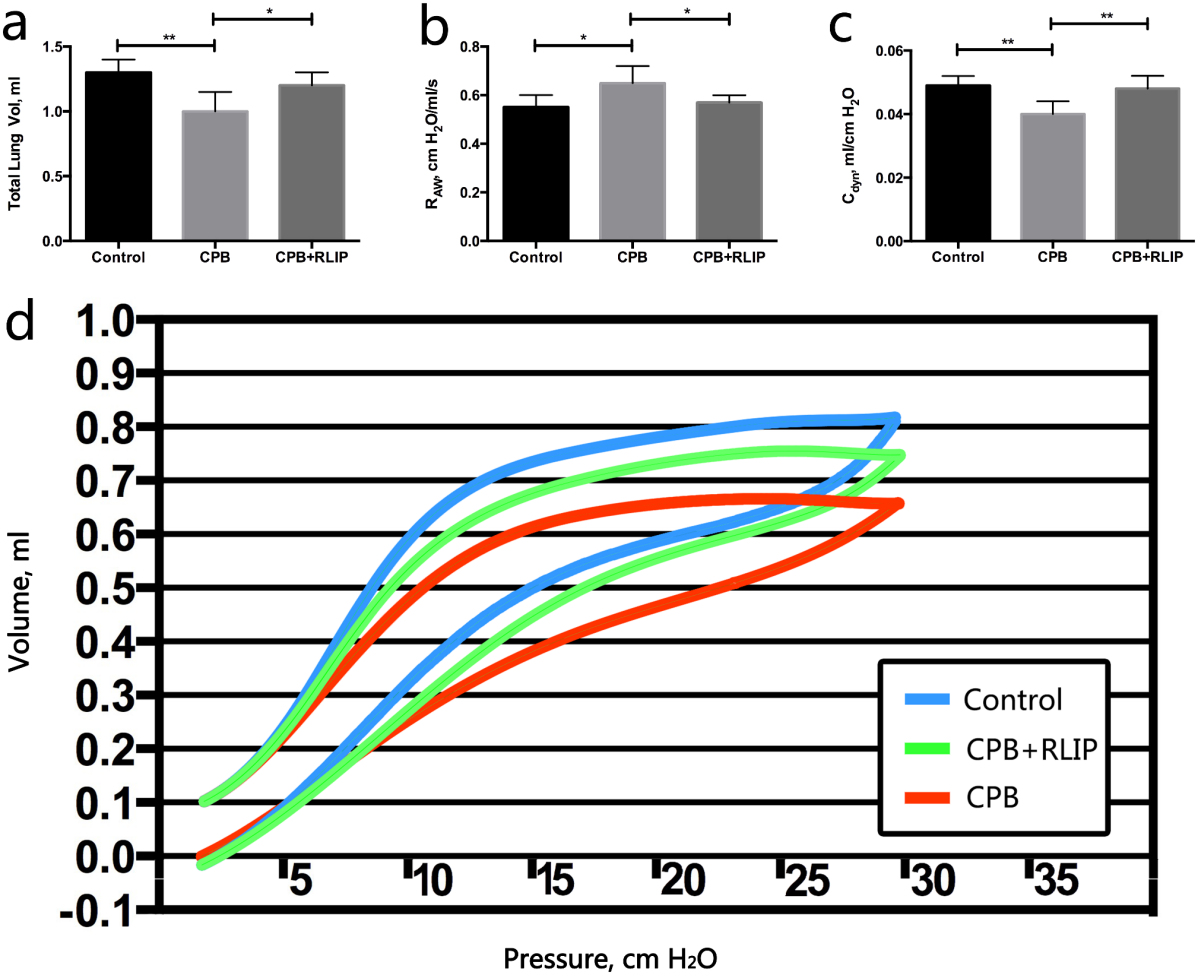
Assessment of the proliferation rates of BASCs isolated from different animal groups by real-time cell analysis.

BASCs were directly isolated from the lungs that were dissected from euthanized rats and monitored on a real-time cell analyzer. The results showed that BASCs obtained from the CPB group proliferated at a much slower rate compared to those isolated from the control group during the 20-h observation period (Fig 3). In contrast, treatment with limb RIPC substantially improved the proliferative capacity of the cells (Fig 3). These results, taken together, suggested that the application of limb RIPC might improve the viability and proliferative capacity of BASCs, leading to enhanced tissue repair in the CPB-injured lungs.

### Limb RIPC exerts its protective effects on lung possibly by altering circulatory microenvironment and up-regulating anti-inflammatory cytokines

**Fig 4:**
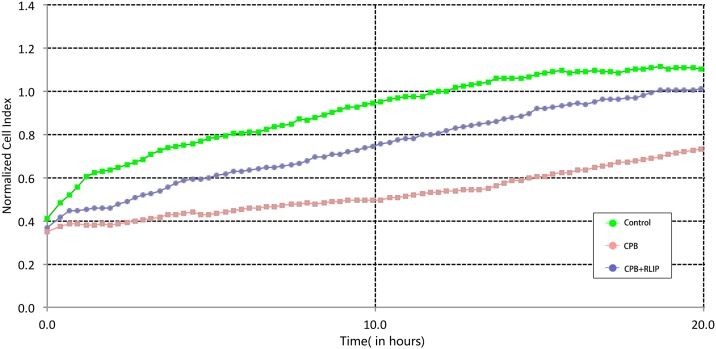
Analysis of serum inflammatory cytokine levels in different animal groups. (A), control; (B), CPB group; (C) treatment group. The levels of different cytokines are expressed as percentages. Each circle represents 1% of the combined level of all nine cytokines assayed.

To probe the possible mechanism through which limb RIPC helped reduce pulmonary injury and the loss of lung function as a result of CPB surgery, we performed blood gas analysis and also measured the levels of various inflammatory cytokines. As summarized in Table 1, the rats that underwent surgical procedure with CPB exhibited significantly lower arterial pH, O_2_ tension and overall arterial pressure than the control group. The treatment with limb RIPC was found to restore these matrices to levels similar to those observed in the control subjects. On the other hand, we also found a decline in the mean arterial CO_2_ tension and the level of serum bicarbonate, as well as a rise in the concentration of serum lactate among the rats in the CPB group compared to those in the control group, although the differences were not statistically significant. The results of the serum cytokine assay revealed that two anti-inflammatory cytokines, IL-4 and IL-10, were significantly down-regulated in the CPB group compared to the control group (Fig 4). As expected, limb RIPC was shown to improve the levels of both cytokines (Fig 4). The results suggested that the pulmonoprotective benefits of limb RIPC were likely associated with its regulation of lung inflammation, particularly its up-regulation of anti-inflammatory cytokines.

**Table 1:** The effect of remote limbo ischemic preconditioning on blood gas analysis.

Variable	Control	CPB+RLIP	CPB	P Value
Aterial pH	7.41 ± 0.05	7.37 ± 0.13*$	7.19 ± 0.08	0.0021
Aterial CO_2_ tension (kPa)	6.4 ± 1.6	6.3 ± 1.5	5.2 ± 1.9	0.4096
Aterial O_2_ tension (kPa; FiO_2_ = 1)	42.1 ± 6.4	41.5 ± 5.5 $	32.3 ± 8.2	0.0426
Serum bicarbonate (mM)	17.8 ± 3.6	17.5 ± 4.5	14.5 ± 2.1	0.2356
Lactate (mM)	4.8 ± 1.4	4.6 ± 1.9	6.0 ± 2.3	0.4087
Mean arterial pressure (mm Hg)	97.5 ± 10.1	90.1 ± 15.3	75.3 ± 7.1	0.0125

Data are expressed as mean ± SD

* Significantly different compared with Control

^$^ Significantly different compared with CPB

## Discussion

The current study sought to evaluate the clinical effects of limb RIPC on reducing lung injury in a rat model of CPB. Our results indicated that rats that were subjected to surgical procedure with CPB sustained significant pulmonary injury as reflected by increased BAL fluid protein content, intra-alveolar infiltration of neutrophils and thickening of the alveolar walls. Correspondingly, the rats in the CPB group exhibited significantly impaired lung function compared to those that did not undergo the procedure. We then demonstrated that limb RIPC could significantly mitigate all of the abovementioned detrimental effects inflicted by CPB and concomitantly ameliorate the respiratory performance of the murine lungs. On a cellular level, we discovered that limb RIPC could improve the proliferation ability of BASCs directly harvested from the CPB-impaired lungs. Further investigation revealed that the pulmonoprotective benefits of limb RIPC were associated with its role in modulating two key anti-inflammatory cytokines, IL-4 and IL-10. The serum levels of both cytokines were down-regulated in the rats with CPB but were partially restored by limb RIPC. These results were consistent with the previously established concept that the propagation of inflammatory cascade leading to the activation and infiltration of various blood cells plays an instrumental role in CPB-induced pulmonary injury [1].

Our finding that limb RIPC exerts its protective effects by up-regulating anti-inflammatory response in lung tissues was echoed by several earlier studies. For example, mice that received RIPC treatment showed alleviated myocardial infarction, together with elevated cardiac IL-10 expression, following artificially induced ischemia-reperfusion [15]. Kim et al. compared the expression levels of pro- and anti-inflammatory cytokines in the hippocampus between Mongolian gerbils that were subjected to lethal ischemia and those that received ischemic preconditioning followed by lethal ischemia [16]. The authors found declined expressions of several anti-inflammatory cytokines, including IL-4 and IL-13, whereas the levels of pro-inflammatory cytokines such as IL-2 and TNF-α were similar in the two animal groups [16]. Interestingly, RIPC was shown to down-regulate the serum levels of TNF-α and IL-6 in a rat model of lipopolysaccharide-induced endotoxemia, suggesting that its protective effects were at least in part due to its direct suppression of pro-inflammatory pathways [17]. As described earlier, local perturbation of serum cytokine levels can spread via the circulatory system to the target organs and trigger changes in various inflammatory pathways that pre-emptively exert a protective effect against future IRI-related injuries. Further studies would be necessary to decipher the downstream targets of the enhanced anti-inflammatory cytokines and provide a better understanding of the regulatory cascades responsible for the clinical benefits of RIPC.

It should be pointed out that several recent clinical studies suggested that the protective effects of RIPC against CPB-induced tissue damage might be more limited than previously thought. In a randomized controlled trial involving cyanosed neonates undergoing surgery with CPB, lower limb RIPC showed no significant regulatory impact on several major renal and cerebral injury markers [18]. This was mirrored in a similar study that applied upper limb RIPC to patients with CPB procedure, where lower incidence of mechanical ventilation, but not improvement in lung function, was observed [19]. Nevertheless, Cheung and colleagues’ early investigation found that lower limb RIPC significantly attenuated cardiac injury, as evidenced by the lower level of troponin, a marker of myocardial infarction, in children undergoing CPB surgery for treatment of heart defects [6]. These conflicting data suggested that the benefits of RIPC might be context- and environment-dependent. For example, it has been argued that the efficacy of RIPC could be reduced in infant hearts by the ample presence of endogenous methionine-enkephalin, which negatively correlates with arterial oxygen concentration and thus could potentially aggravate ischemic symptoms [20]. Clearly, care should be taken with regard to the degree of organ immaturity, treatment dosage and the subject model, in future RIPC experiment designs.

In conclusion, we showed that limb RIPC could significantly alleviate pulmonary injury inflicted by the CPB procedure in a rat model. These findings provided a much-needed addition to the current research efforts that aim to evaluate the protection of RIPC against pulmonary injury caused by CPB. Although changes in inflammatory cytokine levels have been observed in other types of tissues and/or surgeries, our study, to the best of our knowledge, offered the first experimental evidence that increased expression of serum anti-inflammatory cytokines such as IL-4 and IL-10 occurred after limb RIPC in rats receiving surgical procedure with CPB. Further investigation into the molecular mechanism through which the RIPC-enhanced expression of anti-inflammatory cytokines translates into mitigation of tissue injury in lungs and other organs will help medical researchers and practitioners unearth more effective preventative and treatment strategies for CPB-related complications.
